# Cannabinoid receptor 2 (Cb2r) mediates cannabinol (CBN) induced developmental defects in zebrafish

**DOI:** 10.1038/s41598-022-23495-0

**Published:** 2022-11-24

**Authors:** Md Ruhul Amin, Kazi Tanveer Ahmed, Declan William Ali

**Affiliations:** 1 Department of Biological Sciences, University of Alberta, CW-405 Biological Sciences Building, Edmonton, AB T6G 2E9 Canada; 2 Neuroscience and Mental Health Institute, University of Alberta, CW-405 Biological Sciences Building, Edmonton, AB T6G 2E9 Canada

**Keywords:** Neuronal development, Cellular neuroscience, Zebrafish

## Abstract

Of the three primary cannabinoids in cannabis: Δ^9^-Tetrahydrocannabinol (Δ^9^-THC), cannabidiol (CBD) and cannabinol (CBN), very little is known about the actions of CBN, the primary oxidative metabolite of THC. Our goal was to determine if CBN exposure during gastrulation alters embryonic development, and if so, does it act via the canonical cannabinoid receptors. Zebrafish embryos were exposed to CBN during gastrulation and exhibited dose-dependent malformations, increased mortality, decreased locomotion and a reduction in motor neuron branching. Moreover, larva showed a significant reduction in the response to sound stimuli. CBN exposure altered the development of hair cells associated with otic vesicles and the lateral line. Pharmacological block of Cb2rs with AM 630 or JTE 907 prevented many of the CBN-induced developmental defects, while block of Cb1rs with AM 251 or CP 945598 had little or no effect. Altogether we show that embryonic exposure to CBN results in alterations in embryonic growth, neuronal and hair cell development, physiology and behavior via Cb2r-mediated mechanisms.

## Introduction

Cannabis and cannabis-related products are increasingly used for recreational and medicinal purposes^1–5^. Cannabis is also the most commonly used illicit drug during pregnancy^6^ and contains more than 400 chemical compounds, including over 100 different cannabinoids such as delta^9^-tetrahydrocannabinol (Δ^9^-THC), cannabidiol (CBD), and cannabinol (CBN) which is an oxidative metabolite of THC^7^. Δ^9^-THC is the primary psychoactive constituent, whereas the major non-psychoactive constituent is CBD. CBN levels increase during storage and aged cannabis contains more CBN than fresh cannabis^8^. Importantly, CBN was identified as the second most abundant cannabinoid in certain types of cannabis preparations such as hashish and hash oil^9^. But there is very little data on the physiological and developmental effects of CBN.

The endocannabinoid (eCB) system consists of the cannabinoid receptors (Cb1r and Cb2r), their ligands, anandamide (AEA) and 2-arachidonylglycerol (2-AG), and the enzymes that produce and break down AEA and 2-AG. Cb1rs are highly localized to the central nervous system (CNS)^10–12^ while Cb2rs are primarily associated with the peripheral nervous system, the immune system^13^, the intestine, retina and heart^14,15^, with small amounts present in the CNS^16,17^. The eCB system is expressed from very early stages of development shortly following egg fertilization^18,19^. During later stages of neuronal development the eCB system is involved in neurogenic processes such as cell proliferation, maturation and cell survival^19–21^. Cb1r plays an essential role in neuronal development, particularly with regard to axonal pathfinding^22,23^, but a developmental role for Cb2r has not been extensively documented. In zebrafish embryos, Cb2r mRNA is highly expressed along the lateral line organ and knockdown of these receptors alters hair cell development^24,25^. Hair cells of the lateral line are involved in detecting water movement along the trunk and ablation of lateral line hair cells reduces the response rate of zebrafish larvae when they are exposed to accelerated water flow^26^.

In this study, we sought to determine if exposure to CBN during gastrulation, altered embryonic development in zebrafish via actions through the endocannabinoid receptors Cb1r and Cb2r. We chose the time frame of gastrulation because key neurons involved in motor responses (Mauthner cells and primary motor neurons) first appear in zebrafish during gastrulation. In humans this time frame corresponds to approximately the third week after egg fertilization. Our results indicate that embryonic morphology, heart rate, neuronal branching, locomotor responses and hair cell development are adversely affected by exposure to CBN, and that these effects are largely mediated through the Cb2r and not Cb1r.

## Results

### CBN affects gross morphology, heart rate, MN development, locomotion and hair cell development

**Figure 1:**
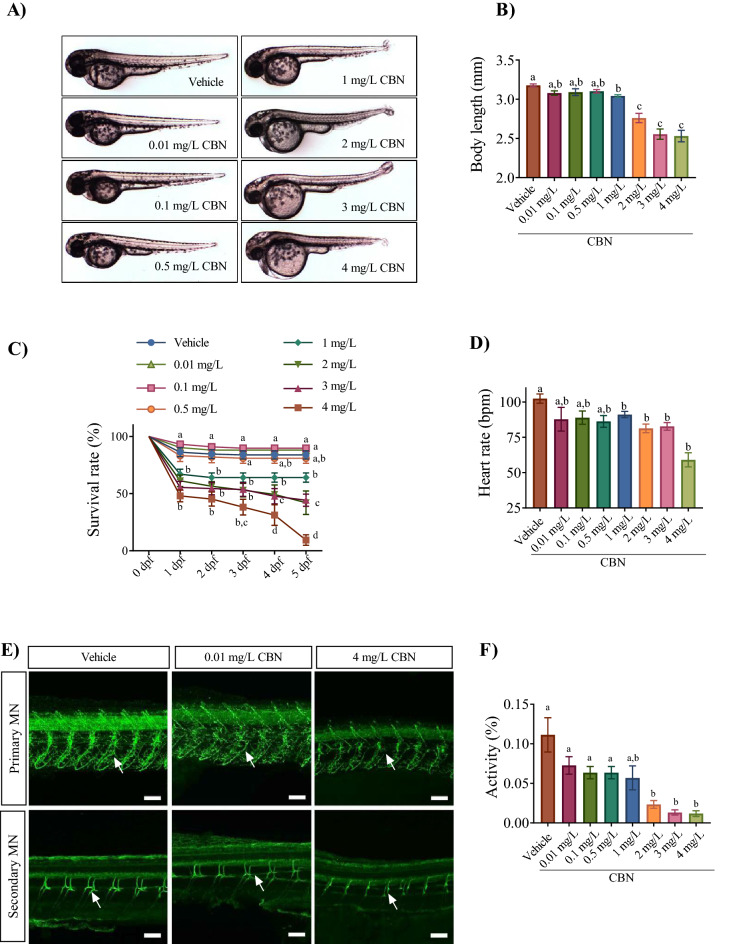
Embryos exposed to CBN during gastrulation show morphological and neuronal deficits. (**A**) Images of embryos exposed to 0.4% methanol (vehicle), 0.01 mg L^−1^, 0.1 mg L^−1^, 0.5 mg L^−1^, 1 mg L^−1^, 2 mg L^−1^, 3 mg L^−1^ and 4 mg L^−1^ CBN (from 5.25 to 10.75 hpf) and then allowed to develop in normal embryo media. Images were taken at 48–52 hpf. (**B**) Bar graph showing the mean body lengths (in mm) of embryos exposed to different media: vehicle control (n = 57), 0.01 mg L^−1^ (n = 61), 0.1 mg L^−1^ (n = 61), 0.5 mg L^−1^ (n = 61), 1 mg L^−1^ (n = 42), 2 mg L^−1^ (n = 30), 3 mg L^−1^ (n = 29) and 4 mg L^−1^ CBN (n = 21) respectively at 2 dpf. N = 3 experiments. (**C**) Line graphs showing the mean percentage of embryos that survived within the first 5 days of development following exposure to different media: vehicle, 0.01 mg L^−1^ CBN, 0.1 mg L^−1^ CBN, 0.5 mg L^−1^ CBN, 1 mg L^−1^ CBN, 2 mg L^−1^ CBN, 3 mg L^−1^ CBN and 4 mg L^−1^ CBN during gastrulation (N = 4 experiments and n = 20 embryos for each treatment). (**D**) Bar graphs of heart rate of embryos exposed to different media: vehicle control (n = 82), 0.01 mg L^−1^ (n = 61), 0.1 mg L^−1^ (n = 23), 0.5 mg L^−1^ (n = 29), 1 mg L^−1^, 2 mg L^−1^, 3 mg L^−1^ and 4 mg L^−1^ CBN (n = 45) respectively; N = 3 experiments. (**E**) Top panel, anti-znp-1 antibody was used to label primary motor neuron axons and their branches (green) in embryos exposed to vehicle control (n = 5), 0.01 mg L^−1^ CBN (n = 7), and 4 mg L^−1^ CBN (n = 7). Branches in a motor axon are indicated with white arrow. Bottom panel, anti-zn8 antibody was used to label secondary MN and their branches (green) in embryos exposed to vehicle control (n = 5), 0.01 mg L^−1^ CBN (n = 7), and 4 mg L^−1^ CBN (n = 7). Scale bar represents 50 µm. Branches in a motor axon are indicated with white arrow. N = 3 experiments. (**F**) Bar graph shows mean activity (%) for embryos exposed to different media: vehicle control (n = 82), 0.01 mg L^−1^ (n = 61), 0.1 mg L^−1^ (n = 23), 0.5 mg L^−1^ (n = 29), 1 mg L^−1^, 2 mg L^−1^, 3 mg L^−1^ and 4 mg L^−1^ CBN (n = 45) respectively. N = 4 experiments. Groups which share the same letter(s) of the alphabet are not statistically different from one another; p < 0.05.

In this study we set out to determine the effect of early CBN exposure on zebrafish development. To do this we exposed embryos during the gastrulation stage (from 5.25 to 10.75 hpf) to concentrations of CBN ranging from 0.01 to 4 mg L^−1^ CBN. We chose this range of concentration because in preliminary experiments, concentrations above 4 mg L^−1^ led to 100% mortality and less than 0.01 mg L^−1^ was ineffective. We found that there were morphological defects in embryos exposed to CBN at concentrations above 0.5 mg L^−1^ (Fig. 1A), but there were no obvious effects at exposure concentrations of 0.5 mg L^−1^ and below. Animals treated with 2, 3 and 4 mg L^−1^ CBN exhibited higher rates of pericardial edema and axial malformations compared with vehicle controls (Supplementary Fig. 1A,B). Embryos exposed to 1, 2, 3 and 4 mg L^−1^ CBN had significantly shorter body lengths compared with controls (Fig. 1B; p < 0.001). Finally, there was a dose dependent effect of CBN on survival and hatching (Fig. 1C; p < 0.001; Supplementary Fig. 1C). Taken together, these data show that at exposure concentrations above 0.5 mg L^−1^ CBN has an impact on embryonic morphological development.

In a previous study we found that exposure to THC and CBD during gastrulation severely affected heart rate in zebrafish embryos^27^. In the present study we obtained similar results for exposure to CBN, such that there was a significant reduction in heart rate for animals treated with CBN at 1 mg L^−1^ or higher (Fig. 1D; p < 0.01) (Supplementary Videos 1, 2).

Next, we asked whether CBN exposure during gastrulation altered motor neuron growth and morphology because zebrafish primary motor neurons first appear between 9 and 11 hpf, during gastrulation when embryos were exposed to CBN. Immunostaining in 2 dpf embryos using the anti-znp1 antibody that targets primary motor neurons showed that exposure to CBN altered the branching pattern of primary motor neurons (Fig. 1E; p < 0.05). For these experiments we restricted our experiments to a low and a high concentration of CBN. Exposure to the lowest concentration of CBN (0.01 mg L^−1^) had no visible effects on primary MN branching while exposure to 4 mg L^−1^ had a severe effect on motor neuron branching (Fig. 1E; n = 10). Additionally, immunostaining secondary motor neurons with anti-zn8 showed that the lateral branching of secondary motor neurons was greatly reduced in the 4 mg L^−1^ CBN group (Fig. 1E; p < 0.05).

We asked if locomotion was impacted and recorded the swimming activity of 5 dpf larva for 60-min. Analysis showed that exposure to CBN in the range of 2–4 mg L^−1^ significantly reduced the mean swimming activity of 5 dpf larvae (Fig. 1F; p < 0.05). Taken together, these data show that exposure to CBN alters morphology, behavior and physiology in developing zebrafish, mainly at exposure concentrations of 1 mg L^−1^ CBN and above.

### CBN exposure affects hair cell development

**Figure 2:**
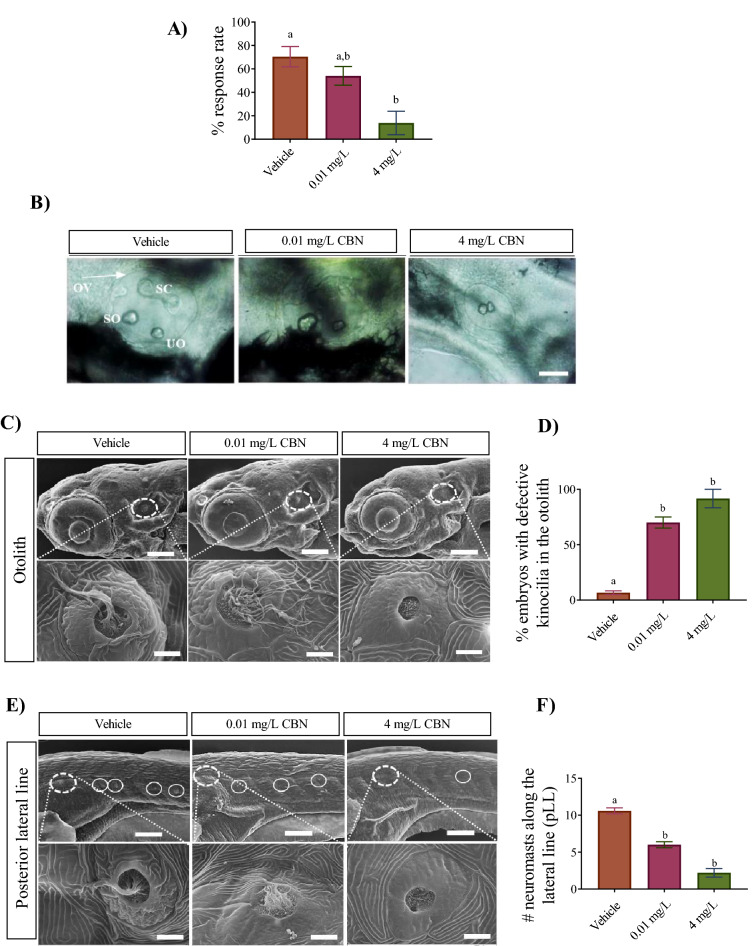
CBN exposure alters the development of hair cells associated with the otoliths and the lateral line. (**A**) Bar graph represents the (%) of embryos that responded to sound/vibration stimuli at 5 dpf. The sound/vibration pulse was delivered to free-swimming embryos from a speaker that was in contact with the base platform and their escape responses were recorded. N = 3 experiments. (**B**) Images of otoloths were taken from 52 hpf old embryos, positioning anterior (head) on right and posterior (tail) on left. The otic vesicles (OV), utricular otolith (UO) and saccular otolith (SO) and semicircular canal (SC) are visible here. N = 3 experiments. Scale bar represents 50 µm. (**C**) Scanning electron microscopy (SEM) images of embryos at 5 dpf. Top rows, images were taken of the otolith at ×500 magnification. Bottom rows, images were taken from inside the otolith at ×10,000 magnification. Kinocilia can be seen emanating from hair cells. Scale bar represents 100 µm (top panel) and 5 µm (bottom panel). N = 3 experiments. (**D**) Bar graph of vehicle controls (n = 6), 0.01 mg L^−1^ (n = 8) and 4 mg L^−1^ (n = 8) groups showing the % of embryos that exhibited changes to the tight bundled structure of kinocilia. N = 3 experiments. (**E**) SEM images of embryos along the posterior lateral line (pLL) at 5 dpf. Top row shows images of vehicle control, 0.01 mg L^−1^ CBN and 4 mg L^−1^ CBN treated groups. Images were taken of the lateral line at ×500 magnification. Bottom row shows the SEM image of the first neuromast along the lateral line. These images were taken at ×10,000 magnification. Scale bar represents 100 µm (top panel) and 5 µm (bottom panel). N = 3 experiments. (**F**) Bar graph showing the number of neuromasts along the posterior lateral line in vehicle controls (n = 6), 0.01 mg L^−1^ (n = 6) and 4 mg L^−1^ (n = 6) groups. N = 3 experiments. Groups which share the same letter(s) of the alphabet are not statistically different from one another; p < 0.05.

Next, we tested whether CBN treatment altered the ability of larvae to respond to an acoustico-vestibular (AV) stimulus. To do this, we provided an auditory/vibration stimulus to 5 dpf larvae and recorded their escape responses. Quantification of the response rate showed that embryos exposed to the lowest concentration of CBN (0.01 mg L^−1^) showed a mild reduction, but there was no significant change in the response rate (Fig. 2A; p < 0.05), whereas embryos treated with the highest dose of CBN (e.g. 4 mg L^−1^) exhibited a major reduction in the response rate of C-bend compared with vehicle controls (Fig. 2A; p < 0.05).

Because CBN treated embryos exhibited reduced responses to sound, we asked whether this altered response might be due to improper development of hair cells associated with the otoliths. Hair cells are crucial for the function of a multitude of tissues and organs^28^ and the cilia associated with hair cells play important roles during CNS development including neurogenesis, neuronal maturation and cell survival^29^. Cilia also play a role in the formation and positioning of otoliths which are biomineralized structures inside the inner ear that lie over patches of sensory hair cells and which are used for hearing and balance in zebrafish. For these experiments, we imaged the otoliths at the time of hatching. The otolith structure appeared normal in embryos treated with the lowest concentration of 0.01 mg L^−1^ CBN, while it was severely altered, and in some cases exhibited completely absent semicircular canals, in animals treated with 4 mg L^−1^ CBN (Fig. 2B). Furthermore, the utricular otoliths (UO) and saccular otoliths (SO) were often fused in animals exposed to 4 mg L^−1^ CBN (Fig. 2B). Next, we sought to determine whether CBN exposure affected the development of hair cells associated with the otoliths and the lateral line. SEM images showed disorganization of otolith kinocilia (Fig. 2C,D) and reduced numbers of neuromasts along the posterior lateral line (Fig. 2E,F; n = 6) in animals that were treated with the lowest concentration of CBN (0.01 mg L^−1^). About 53 ± 8% of the embryos had altered cilia in their otoliths compared to controls in which only 7 ± 2% of the animals had unbundled and disorganized cilia (Fig. 2D; p < 0.01). For comparison, the kinocilia in control animals appeared well-organized into tight bundles. Animals treated with the highest doses of CBN (4 mg L^−1^) exhibited severe changes to kinocilia in the otoliths and neuromasts of the lateral line (Fig. 2C,E). For instance, kinocilia were completely absent inside the otolith as shown in Fig. 2C. Almost every embryo examined (92 ± 8%) showed defective features of their hair cells compared with controls in which only 7 ± 2% of the animals exhibited altered hair cell features. (Fig. 2D, p < 0.01). Furthermore, there were significantly high numbers of absent neuromasts along pLL in animals treated with 4 mg L^−1^ CBN as shown in Fig. 2E,F (p < 0.05). These data show that very small concentrations of CBN have the ability to alter hair cell and cilia development.

### Blocking Cb2r activity rescue morphology

Next, we set out to determine if the cannabinoid receptors, Cb1r and Cb2r were involved in mediating the CBN-induced developmental defects. To do this, we used pharmacological blockers of Cb1r (AM251 and CP94) and Cb2r (AM630 and JTE907) to block the activity of these receptors in the presence of CBN. The concentration of Cb1r and Cb2r antagonists (10 nM and 10 µM respectively) were chosen based on dose response studies (Supplementary Figs. 2–4). For these experiments, we used a concentration of CBN (3 mg L^−1^) that had a significant effect on the embryos, so that inhibition of the effects of CBN could be clearly determined. Moreover, we used two different antagonists for each receptor type to confirm our results in case receptor specificity was an issue.

**Figure 3:**
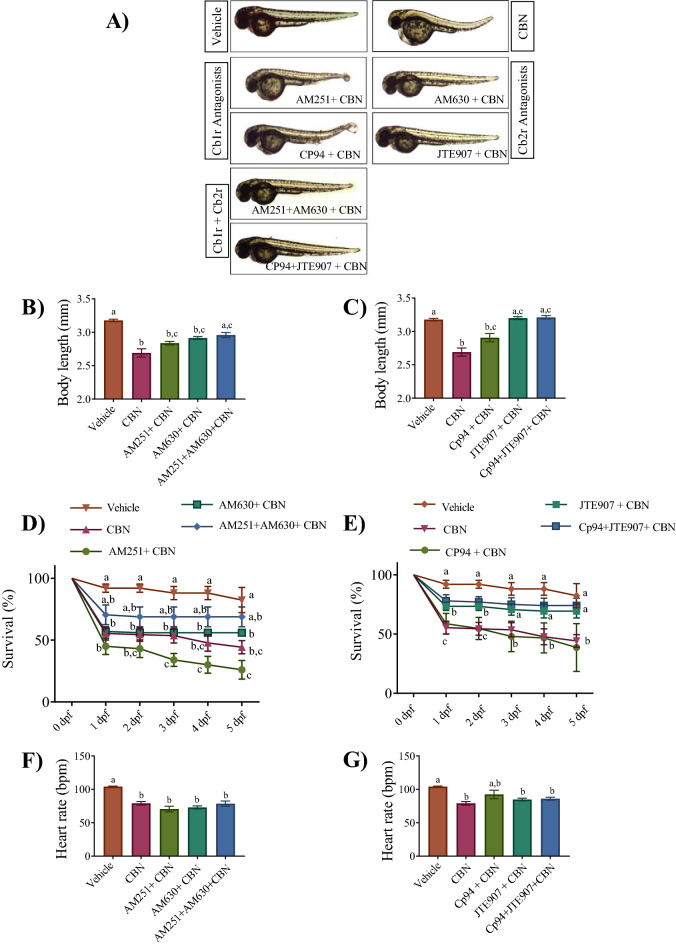
Blocking Cb2r prevents the CBN-induced deficits in morphology and survival. (**A**) Images of embryos at 2 dpf in the presence and absence of Cb1r and Cb2r antagonists with CBN. N = 3 experiments. (**B**) Bar graph shows the mean body length (mm) of embryos for one set of antagonists (AM251 and AM630 for Cb1r and Cb2r respectively) in vehicle control (n = 57), 3 mg L^−1^ CBN (n = 29), AM251 10 nm + 3 mg L^−1^ CBN (n = 21), AM630 10 µm + 3 mg L^−1^ CBN (n = 21) and AM251 10 nm + AM630 10 µm + 3 mg L^−1^ CBN (n = 36) respectively at 2 dpf (N = 3 experiments). (**C**) Bar graph shows the mean body length (mm) of embryos for the second set of antagonists (Cp94 and JTE907 for Cb1r and Cb2r respectively). The bars represent vehicle control (n = 57), 3 mg L^−1^ CBN (n = 29), CP94 10 nm + 3 mg L^−1^ CBN (n = 31), JTE907 10 µm + 3 mg L^−1^ CBN (n = 234) and Cp94 10 nm + JTE907 10 µm + 3 mg L^−1^ CBN (n = 31) at 2 dpf. N = 3 experiments. (**D**) Line graph showing the percentage of embryos that survived within the first 5 days of development following co-exposure of the first set of antagonists and CBN. Treatments are vehicle control, 3 mg L^−1^ CBN, AM251 10 nm + 3 mg L^−1^ CBN, AM630 10 µm + 3 mg L^−1^ CBN and AM251 10 nm + AM630 10 µm + 3 mg L^−1^ CBN (N = 4 experiments and n = 25 embryos for each experiment). (**E**) Line graph showing the percentage of embryos that survived within the first 5 days of development following co-exposure of the second set of antagonists and CBN. Treatments are vehicle control, 3 mg L^−1^ CBN, CP94 10 nm + 3 mg L^−1^ CBN, JTE907 10 µm + 3 mg L^−1^ CBN and Cp94 10 nm + JTE907 10 µm + 3 mg L^−1^ CBN (N = 4 experiments and n = 25 embryos for each treatment). (**F**) Bar graphs show the mean heart rate in beats per minute (bpm) at 2 dpf for vehicle control (n = 35), 3 mg L^−1^ CBN (n = 59), AM251 10 nm + 3 mg L^−1^ CBN (n = 32), AM630 10 µm + 3 mg L^−1^ CBN (n = 33) and AM251 10 nm + AM630 10 µm + 3 mg L^−1^ CBN (n = 20) respectively. N = 4 experiments. (**G**) Bar graphs show heart rate as beats per minute (bpm) at 2 dpf for vehicle control (n = 35), 3 mg L^−1^ CBN (n = 59), CP94 10 nm + 3 mg L^−1^ CBN (n = 21), JTE907 10 µm + 3 mg L^−1^ CBN (n = 25) and Cp94 10 nm + JTE907 10 µm + 3 mg L^−1^ CBN (n = 20) respectively. N = 4 experiments. Groups which share the same letter(s) of the alphabet are not statistically different from one another; p < 0.05.

CBN treated animals had shorter bodies (2.69 ± 0.06 mm) compared with controls (3.78 ± 0.01 mm) (Fig. 3A–C; p < 0.01). Co-treatment of CBN with either of the Cb1r antagonists, AM251 or CP94, had small but non-significant effects on the reduction of body length induced by CBN exposure (Fig. 3A–C; p < 0.01). In contrast, co-treatment of either of the Cb2r antagonists, AM630 or JTE907, resulted in a very strong or complete block of the effects of CBN (Fig. 3A–C; p < 0.01).

When examining animal mortality, we found that blocking Cb1r activity with either AM251 or CP94 did not inhibit CBN-induced mortality by 5 dpf (Fig. 3D,E), indicating that the effects of CBN were not due to activation of Cb1r. However, inhibition of Cb2r activity with either AM630 or JTE907 resulted in a significant reduction in mortality (Fig. 3D,E; p < 0.01). Animals co-treated with both Cb1r and Cb2r antagonists showed no significant differences from animals treated with Cb2r antagonists + CBN alone (Fig. 3A–E). Taken together, these results suggest that CBN alters aspects of morphology and survival through activation of Cb2rs but not Cb1rs.

The effect of CBN on heart rate was not prevented by blocking either Cb1r or Cb2r (Fig. 3F,G; p < 0.05). Interestingly, heart morphology appears largely normal in the presence of Cb2r antagonists (Supplementary Video 3), but the mean heart rate remains slower than controls, suggesting that CBN works through Cb2r to alter some aspects of heart development but not all. Overall, the effects on cardiac activity appear to occur via receptors and intracellular systems that are distinct from the canonical cannabinoid receptor pathways.

### CBN induces alterations in MN branching via Cb2rs

**Figure 4:**
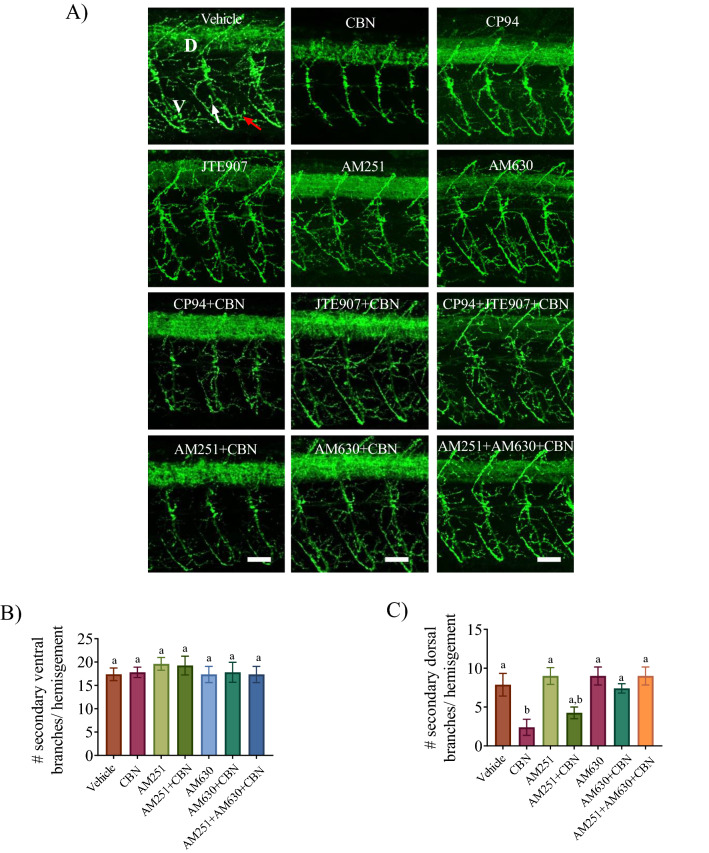
Combined exposure of Cb2r and CBN inhibits CBN-induced changes in primary motor neuron branching. (**A**) Znp-1 (green) antibody was used to label primary motor neuron axons and their branches in vehicle control (n = 7), 3 mg L^−1^ CBN (n = 9), AM251 10 nm + 3 mg L^−1^ CBN (n = 8), CP94 10 nm + 3 mg L^−1^ CBN (n = 9), AM630 10 µm + 3 mg L^−1^ CBN (n = 9), JTE907 10 µm + 3 mg L^−1^ CBN (n = 8), AM251 10 nm + AM630 10 µm + 3 mg L^−1^ CBN (n = 5) and Cp94 10 nm + JTE907 10 µm + 3 mg L^−1^ CBN (n = 6). Dorsal and ventral branches in a motor axon are indicated with (D) and (V) respectively, and the white arrow points to secondary branches in the vehicle image. N = 3 experiments; Scale bar represents 25 µm. (**B**,**C**) Bar graph shows the number of secondary branches in the dorsal and ventral regions of the trunk. N = 3 experiments. Groups which share the same letter(s) of the alphabet are not statistically different from one another; p < 0.05.

Next, we tested if blocking either of the Cb1 or Cb2 receptors prevented the CBN-induced alterations in motor neuron morphology. Block of Cb1r with either AM251 or CP94, did not significantly prevent the CBN-induced deficits in primary motor neuron branching (Fig. 4; p > 0.05). In contrast, block of Cb2r activity with either AM630 or JTE907 resulted in primary motor neuron branching patterns that were very similar to vehicle controls (Fig. 4A). These observations were confirmed through quantification of the number of secondary branches emanating from the main motor neuron axon as shown in Fig. 4B,C. Detailed quantification was performed for one set of antagonists for each of the receptors: AM251 for the Cb1r and AM630 for the Cb2r (Supplementary Fig. 5A–E). We analyzed the ventral and dorsal branching separately because this could be done with confidence. Examination of secondary branching in the ventral regions showed that there were no differences between treatment groups (Fig. 4B). However, CBN treatment caused a significant reduction in the number of secondary branches in the dorsal trunk from 8 ± 1 in the control animals to 2 ± 1 branches after CBN treatment (Fig. 4C; p < 0.05). Co-exposure of AM251 with CBN, had no significant effect on the number of branches (Fig. 4C), whereas animals co-treated with the Cb2r blocker, AM630 and CBN had secondary dorsal branch numbers (7 ± 1) that were similar to controls 8 ± 1 (Fig. 4C; p < 0.05). These results suggest that CBN alters neuronal development of motor neurons via activation of Cb2rs.

### Co-exposure of CBN and Cb2r antagonists prevent locomotor deficits

**Figure 5:**
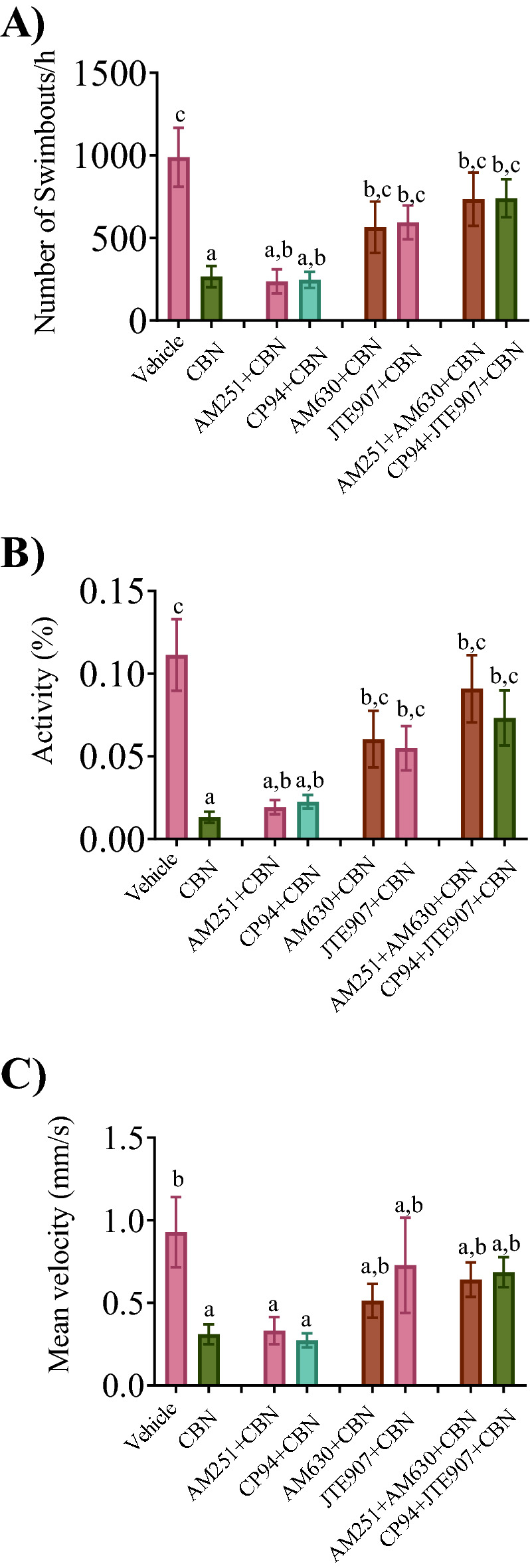
Combined exposure of Cb2r with CBN prevents the CBN-induced deficits in locomotion. The free swimming movement of the fish was recorded for 60 min and the recorded video was analyzed later. Tracing was recorded at 5 dpf for vehicle control (n = 18), 3 mg L^−1^ CBN (n = 25), AM251 10 nm + 3 mg L^−1^ CBN (n = 37), CP94 10 nm + 3 mg L^−1^ CBN (n = 31), AM630 10 µm + 3 mg L^−1^ CBN (n = 32), JTE907 10 µm + 3 mg L^−1^ CBN (n = 37), AM251 10 nm + AM630 10 µm + 3 mg L^−1^ CBN (n = 36) and Cp94 10 nm + JTE907 10 µm + 3 mg L^−1^ CBN (n = 40) respectively. N = 4 experiments. (**A**) Bar graph shows the mean frequency of swim bouts within 1 h. (**B**) Bar graph shows the mean swimming activity (%) in 1 h. (**C**) Bar graph shows the mean swimming velocity (mm s^−1^) over a 1 h time period. Groups which share the same letter(s) of the alphabet are not statistically different from one another; p < 0.05.

5 dpf larva exposed to CBN (3 mg L^−1^) during gastrulation exhibited greatly reduced levels of movement compared with vehicle treated animals. Co-exposure of either of the Cb1r antagonists (AM251 and CP94) with CBN did not prevent the effects of CBN treatment alone (Fig. 5). However, co-exposure of either of the Cb2r antagonists (AM630 or JTE907) with CBN significantly inhibited the effects of CBN treatment on the number of swim bouts per hour, activity and velocity compared with vehicle controls (Fig. 5; p < 0.05). Finally, animals treated with combinations of Cb1r and Cb2r blockers exhibited mean numbers of swim bouts and swimming activity that were comparable to those obtained when blocking Cb2r alone (Fig. 5; p < 0.05). Taken together our findings show that the effect of CBN treatment on locomotor activity is mediated via actions on Cb2rs and not Cb1rs.

### Pharmacological blocking of Cb2r inhibits CBN-induced alteration of hair cell development

**Figure 6:**
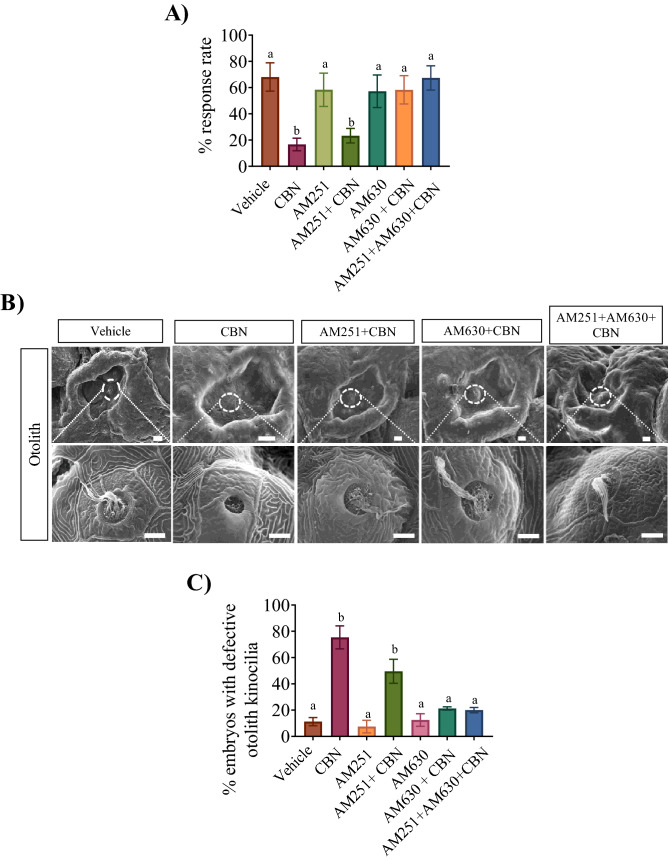
Blocking Cb1r and Cb2r prevents the CBN-induced changes in otolith hair cell development. (**A**) Bar graph shows the mean percentage of animals that responded to sound/vibration stimulus in the presence or absence of receptor blockers and CBN in 5 dpf larvae. The response to sound stimuli (C-bend) was recorded following exposure to sound and analyzed later for calculating response rate (%). N = 3 experiments. (**B**) SEM images were taken of the head i.e. anterior lateral line (aLL). (**B**) Images were taken at ×500 magnification for 3 mg L^−1^ CBN (n = 5), AM251 10 nm + 3 mg L^−1^ CBN (n = 5), AM630 10 µm + 3 mg L^−1^ CBN (n = 5) and AM251 10 nm + AM630 10 µm + 3 mg L^−1^ CBN (n = 5) respectively. Scale bar represents 20 µm. (**C**) Bar graph showing the quantification of the percentage of the embryos that exhibited deformities in the kinocilia associated with the otoliths. Groups which share the same letter(s) of the alphabet are not statistically different from one another; p < 0.05.

We asked whether block of Cb1r or Cb2r prevented the CBN-induced deficits in the acoustico-vestibular escape response. Analysis of escape responses showed that CBN exposure (3 mg L^−1^) significantly reduced the response rate in 5 dpf larva from a mean value of 68 ± 11% in vehicle control animals to 17 ± 5% in the CBN treated group (Fig. 6A; p < 0.05). Either AM630 (Cb2r antagonist) or AM251 + AM630 co-exposure with CBN prevented the CBN-induced deficits in the escape response rate, which was 58 ± 11% and 67 ± 9% respectively (Fig. 6A; p < 0.05). Application of the Cb1r antagonist, AM251 did not prevent the CBN-induced deficits in escape responses (Fig. 6A; p > 0.05).

Next, blocking the activity of either Cb1r or Cb2r partially prevented the CBN-induced alterations of hair cell development associated with the otoliths (Fig. 6 and Supplemental Fig. 5F–I). Co-exposure of AM630 or AM251 + AM630 with CBN largely prevented the CBN-induced effects in the otoliths (Fig. 6B). Further quantification showed that co-exposure of AM630 + CBN or AM251 + AM630 with CBN resulted in a reduction in the number embryos with defective kinocilia within their otoliths compared to CBN treated animals (Fig. 6C; p > 0.05). In contrast, co-treatment of AM251 alone with CBN did not prevent the deficits induced by CBN (Fig. 6).

**Figure 7:**
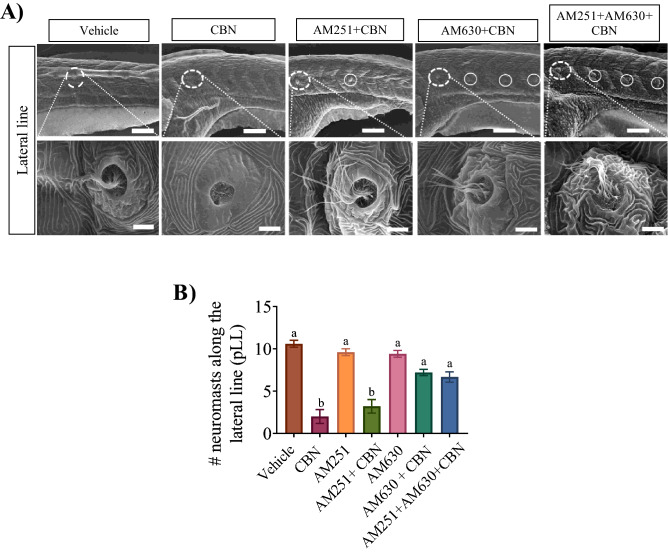
Blocking Cb1r and Cb2r activity prevents the CBN-induced changes in hair cell development along the pLL. (**A**) Images are taken along the pLL at ×500 magnification for 3 mg L^−1^ CBN (n = 5), AM251 10 nm + 3 mg L^−1^ CBN (n = 5), AM630 10 µm + 3 mg L^−1^ CBN (n = 5) and AM251 10 nm + AM630 10 µm + 3 mg L^−1^ CBN (n = 5). Scale bar represents 100 µm for the top row. N = 3 experiments. The bottom row shows higher magnification images of the pLL (×10,000 magnification). Scale bar represents 5 µm. (**I**) Bar graph showing the quantification of the percentage of the embryos that exhibited deformities in their hair cell along pLL. N = 3 experiments. Groups which share the same letter(s) of the alphabet are not statistically different from one another; p < 0.05.

Finally, we tested if blocking Cb2r prevented the CBN-induced effects on hair cells associated with the lateral line (Fig. 7). We found that co-exposure of AM630 or AM251 + AM630 with CBN largely prevented the CBN-induced effects along the posterior lateral line (Fig. 7A). Quantification of the number of neuromasts along the pLL showed that the Cb2r blocker AM630 prevented the CBN-induced reduction in neuromasts (Fig. 7B; p > 0.05). Taken together, these findings indicate that the CBN-induced alterations in hair cell morphology are primarily due to activation of Cb2rs.

## Discussion

The recent legalization of cannabis in various parts of the world has highlighted the paucity of information on the effects of cannabinoids during very early development. Several studies have examined the effects of THC and CBD on developing organisms^27,30–32^, but information on the effect of CBN during early development is scarce. This is in part due to the low concentration of CBN in freshly prepared cannabis; however, as THC oxidizes, the concentration of CBN increases and over time may build up to significantly high concentrations^8,33^. Recent findings show that after a year of dark-storage, almost 22% of the THC content degraded to CBN with a transformation rate of 4.4% every three months^8^. Therefore, improper handling and long-term storage of cannabis will result in increased levels of CBN. In the present study, we examined the effects of a range of CBN concentrations (0.01–4 mg L^−1^) on zebrafish development. To our knowledge, this is the first study to report the effect of CBN on neuronal development in embryos.

Our findings indicate that doses of CBN in the range of 0.01–0.5 mg L^−1^ did not significantly alter the general morphology or branching patterns of motor neurons, whereas higher concentrations (1–4 mg L^−1^) altered features such as morphology, body length, survival, heart rate and neuronal structure. We rationalized our use of these concentrations as follows: human subjects who smoked cannabis cigarettes that contained about 0.2% CBN, experienced concentrations of CBN in their blood plasma as high as 2.4 µg L^−1^^34^. Since the CBN content of some cannabis strains can be as high as 2–5%^9^, it suggests that the blood plasma concentration of CBN can be up to 0.024–0.05 mg L^−1^. Thus, when combined with a 0.1–10% toxicant permeability rate across the zebrafish chorion, we estimated that exposure of the chorion to 0.01–4 mg L^−1^ covered a wide range of relevant CBN concentrations.

In zebrafish, cannabinoid receptors are present from very early stages of development. Quantification of mRNA show that both Cb1r and Cb2rs are present from as early as 1 h post fertilization (hpf), well before gastrulation begins, but the expression patterns are different such that the expression of Cb2r is high in the first day of development and then declines, whereas the expression of Cb1r is low but then increases after 24 h^35,36^. Both receptors (Cb1r and Cb2r) mediate their cellular activity through G-protein dependent (G_s_, G_i/o_, G_q/11_ and G_12/13_) and G-protein independent pathways (G-protein receptor kinases and ion channels)^37–39^. Traditionally, they are coupled to G_i/o_ proteins which typically lead to inhibition of multiple downstream events such as inhibition of adenylyl cyclase, a reduction in voltage gated calcium channel activity, and activation of G-protein gated inwardly rectifying potassium channels (GIRK) and mitogen-activated protein kinases (MAPK)^40,41^.

Low concentrations of CBN (0.01 mg L^−1^) did not alter the branching patterns of motor neurons whereas the higher concentrations of CBN (4 mg L^−1^) severely affected motor neuron development. The primary motor neurons are born towards the end of gastrulation, around 9–11 hpf and their axons soon extend to skeletal muscles^42^. In contrast, secondary motor neurons first appear around 15 hpf until 25 hpf^43^. In each hemi-segment, there are 3–4 primary motor neurons and up to 20–24 secondary motor neurons. Therefore, primary motor neuron cell bodies are present at the time of exposure and may be directly impacted by CBN. Interestingly, the secondary motor neuron branching was also affected by higher doses of CBN even though they were born 5–6 h after the end of the exposure period, suggesting that CBN may remain associated with lipid tissue after washout. In our study blocking Cb1rs partially prevented the alterations in primary motor neuron branching whereas blocking Cb2rs almost completely prevented the changes in motor neuron branching. Block of Cb1r and Cb2r together resulted in similar results obtained when blocking only Cb2r implying that Cb2r activity is primarily involved in mediating the effects of CBN, and not Cb1rs. Our studies on locomotion showed that inhibiting Cb2r activity prevented the reduced swimming activity of CBN-treated embryos. We only observed a slight improvement in the swimming performance of embryos in the presence of Cb1r blockers and combined blocking of Cb1r and Cb2r did not produce additive or synergistic effects. Taken together, our results suggest that during gastrulation CBN preferentially interacts with Cb2rs to alter motor neuron growth and locomotion. These findings are consistent with the higher relative expression of Cb2r in the first 24 h of development compared with Cb1r, as well as with the relative higher affinity of CBN for Cb2rs compared with Cb1r^35^. These findings also highlight the possibility that Cb2rs within the early CNS may be involved in some aspects of locomotor development.

One of our most provocative findings is the effect of CBN exposure on hair cell development. Our result suggests that exposure to CBN affects the development of hair cells associated with the inner ear and the lateral line system, even when exposed to very low concentrations of CBN (0.01 mg L^−1^). When exposed to the higher concentrations of CBN (4 mg L^−1^), the development of hair cells was severely impacted. The structure, development and the function of hair cells are conserved between fish and humans. Exposure to lower concentrations of CBN (0.01 mg L^−1^) resulted in splayed hair cells that is indicative of the downregulation of structural proteins. Co-exposure of embryos to CBN and the Cb2r antagonist (AM630) significantly improved the response rate of CBN treated embryos to sound. Blocking Cb2r activity also prevented the deficits in the morphology of hair cells associated with the otoliths and the lateral line (pLL). Interestingly, Cb2rs have been shown to be highly expressed in hair cells, which aligns with our current findings^24^.

Arguably, our most critical finding is that a concentration of CBN as low as 0.01 mg L^−1^ (and possibly even lower if one takes into account the limited diffusion through the chorion), has a measurable impact on cilia development. A number of signaling pathways such as those involving sonic hedgehog (shh), wnt and notch are involved in hair cell proliferation, development and regeneration^44^. Shh in particular plays an important role during hair cell development, partly via activation of smoothened (Smo), a G-protein coupled receptor^45,46^. In fact, several studies report a role of Cb2r activation in hair cell development in rodents and zebrafish^24,25^ and recent reports point to a potential heterodimerization of smo and cannabinoid receptors^46^. In these models, Smo acts as a co-receptor with Cb2r to activate the sonic hedgehog (Shh) pathway through Gαi, which inhibits adenylate cyclase (AC), cAMP production and ultimately reduces PKA activity. Inhibition of PKA maintains Gli transcription factors in their activator forms (Gli A), thereby facilitating gene transcription for normal development of hair cells^28,47^. Early exposure to CBN may impact this pathway by either inhibiting smo activity, which results in an upregulation of PKA and an accumulation of a Gli repressor (Gli R)^46,48^, or by binding to Cb2r, thereby stimulating PKA activity and increasing the Gli repressor, Gli R. In either case, the potentially harmful effect of CBN exposure to developing hair cells is of major importance precisely because cilia play critical developmental roles in brain function, retinal, hearing and kidney function.

Overall, our results suggest that exposure to CBN very early in life may alter the embryonic development of zebrafish in Cb2r-dependent manner. We recognize that many of the more obvious effects were obtained at the higher exposure concentrations, but the effects of very low doses of CBN on hair cells and cilia are concerning. While our exposure paradigm was very brief, these results should be interpreted with care. The impact of cannabinoids such as CBN on developing embryos is something that must be taken into account when making decisions around family planning and it is clear that more research needs to be done to fully understand the effect of CBN exposure on developing organisms as even brief exposure may have an impact on embryonic health and development.

## Methods

### Animal care and exposure to CBN

The fish used in this study were wild type zebrafish (*Danio rerio*) embryos of the Tubingen Longfin (TL) strain that were maintained at the University of Alberta Aquatic Facility. All animal housing and experimental procedures were approved by the Animal Care and Use Committee at the University of Alberta (AUP #00000816) and adhered to the Canadian Council on Animal Care guidelines for humane animal use. The reporting in this manuscript follows the recommendations in the ARRIVE guidelines. For breeding, 3–5 adults, usually consisting of 3 females and 2 males, were placed in breeding tanks the evening before eggs were required. The following morning, fertilized eggs were collected from the breeding tanks, usually within 30 min of fertilization. Embryos and larvae were housed in incubators on a 12 h light/dark cycle, and set at 28.5 °C. Embryos were exposed to egg water (EW; 60 mg mL^−1^ Instant Ocean) containing CBN (0.01–4 mg L^−1^ diluted from a stock solution in methanol obtained from Sigma) or equivalent amounts of methanol or combination of appropriate drugs during the period of gastrulation, which occurs between 5.25 and 10.75 hpf. The highest methanol concentrations used in vehicle controls and in any of the treatments was 0.4%. The exposure medium was then replaced at 10.75 hpf with 25 mL of fresh EW. Embryos were washed several times in EW and then incubated in fresh EW until further experiments at 48 hpf. Antagonists of Cb_1_r (AM251 (Seleckchem, cat # S2819), CP945598 (Apexbio, Cat #1435; referred as CP94)) and Cb_2_r [AM630 (Tocris Bioscience, cat #1120), JTE907 (Tocris Bioscience, cat #2479)] were used in some experiments. For immunohistochemical studies, pigment formation was blocked by adding 0.003% phenylthiourea (PTU) dissolved in egg water at 24 hpf. All protocols were carried out in compliance with guidelines described by the Canadian Council for Animal Care (CCAC) and the University of Alberta.

### Imaging and morphology

Embryos were imaged at 2 dpf using a Lumenera Infinity2-1R colour microscope camera mounted on a Leica stereomicroscope. Embryos were placed in a 16-well plate with one embryo per well and were anesthetized in 0.02% MS222. Morphological observations were performed using a dissecting microscope; embryos were placed in a 16-well plate with one embryo per well and anesthetized in 0.02% MS222. Measurements of embryo length were done using a microscope eyepiece equipped with a micrometer.

### Immunohistochemistry

Embryos at 2 days post fertilization (dpf) were fixed in 2% paraformaldehyde for 1–2 h and washed with 0.1 M phosphate buffered saline (PBS) every 15 min for 2 h. The preparations were then permeabilized for 30 min in 4% Triton-X 100 containing 2% BSA and 10% goat serum. Tissues were incubated for 48 h at 4 °C in either mouse monoclonal anti-Znp-1 which targets primary motor axons^49^, or mouse monoclonal anti-Zn-8^49^ (DSHB), which identifies secondary motor axons^50^. All primary antibodies were diluted at 1:250 in PBS. Tissues were washed in PBS twice every 15 min for 2–3 h and then incubated for 4 h at room temperature in the secondary antibody, Alexa Fluor^®^ 488 goat anti-mouse IgG, (Molecular Probes, Life Technologies), at a dilution of 1:1000. The embryos were then washed for 7 h with PBS and mounted in MOWIOL mounting media. For labelling of nAChRs, embryos at 2 dpf were permeabilized as previously stated and incubated with 100 nM Alexa-488 conjugated α-bungarotoxin (Molecular Probes, Invitrogen) for 4 h at room temperature. Embryos were then washed for 7 h with PBS and mounted in MOWIOL mounting media. All embryos were imaged on a Zeiss LSM confocal microscope and photographed under a 40 × objective. Images were compiled using Zeiss LSM Image Browser software and are shown as maximum intensity z-stack compilations. For primary motor axon branches, Image J neurite tracer plug-in (Fig. 4) was used to count the number of branches per hemi segment.

### Scanning electron microscopy (SEM)

At 5 dpf, embryos were collected into fixative-2.5% glutaraldehyde, 2% paraformaldehyde in 0.1 M phosphate buffer fixative, pH 7.4. The embryos were then washed in buffer and dehydrated through a graded series of ethanol. After the ethanol series the embryos went through a series of ethanol: Hexamethyldisilazane (HMDS) mixtures, ending with pure HMDS. From HMDS, the embryos were air dried overnight. Embryos were mounted on SEM stubs and sputter-coated with gold–palladium. The samples were examined in a Zeiss EVO 10 scanning electron microscope using acceleration voltage of 15 kV.

### Free swimming (locomotion at 5 dpf)

To track behavioral activities, such as velocity, swim bouts and activity, larvae at 5 dpf were placed in a 96 well plate and then followed procedures similar to other. Larvae were placed gently into the center of wells containing 150 µL egg water, pH 7.0 and 48 wells were used each time from a 96 well plate in our study (Costar #3599). For these swimming experiments we tested animals that showed no obvious morphological deficits. Prior to video recording, larvae were acclimated in the well plate for 60 min. Plates were placed on top of an infrared backlight source and a Basler GenlCaM (Basler acA 1300-60) scanning camera with a 75 mm f2.8 C-mount lens, provided by Noldus (Wageningen, Netherlands) was used for individual larval movement tracking.

EthoVision_®_ XT-11.5 software (Noldus) was used to quantify activity (%), velocity (mm s^−1^), swim bouts frequency and cumulative duration of swim bouts for one hour. To exclude background noise, ≥ 0.2 mm was defined as active movement. Activity was defined as % pixel change within a corresponding well between samples (motion was captured by taking 25 samples/frames per second). The absolute values of mean activity may appear small due to pixel percentage in our region of interest (a well) that are changing at any time is small and bursts of activity may occur such that total movement is short within any given minute.

### Statistics

All values are reported as means ± SEM (standard error of the mean). Significance was determined using a one-way ANOVA followed by a Tukey post-hoc multiple comparisons test where appropriate (p < 0.05). When normality tests failed, a Kruskal–Wallis one-way ANOVA on ranks was performed followed by a Dunn’s multiple comparisons test (p < 0.05). Statistical analysis was done using the statistical software built into GraphPad prism.

## Supplementary Information

Supplementary Information.

Supplementary Video 1.

Supplementary Video 2.

Supplementary Video 3.

## Data Availability

The data that support the findings of this study are available from the corresponding author upon reasonable request. Some data may not be made available because of privacy or ethical restrictions.
