# Supplementary material for: Cannabinoid receptor 2 (Cb2r) mediates cannabinol (CBN) induced developmental defects in zebrafish
Source: Sci Rep. 2022 Nov 24;12:20251. doi: 10.1038/s41598-022-23495-0 (PMC9691751; doi:10.1038/s41598-022-23495-0)
Supplement: Supplementary file 1 — Supplementary Information. [file 41598_2022_23495_MOESM1_ESM.docx]

**Supplement Figure legends**

**Figure 1: Effect of CBN exposure during gastrulation on zebrafish embryos.** (A) Bar graph showing the pericardial edema (%) of embryos exposed to different media: vehicle control (n = 57), 0.01 mg/L CBN (n = 61), 0.1 mg/L CBN (n = 61), 0.5 mg/L CBN (n = 61), 1 mg/L CBN (n = 42), 2 mg/L CBN (n = 30), 3 mg/L CBN (n = 29) and 4 mg/L CBN (n = 21) respectively at 2 dpf. (B) Bar graph showing the axial malformation (%) of embryos exposed to different media: vehicle control (n = 82), 0.01 mg/L CBN (n = 61), 0.1 mg/L CBN (n=23), 0.5 mg/L CBN (n=29), 1 mg/L CBN, 2 mg/L CBN, 3 mg/L CBN and 4 mg/L CBN (n=45) respectively. (C) Line graphs showing the percentage of embryos that hatched within the first 5 days of development following exposure to different media: vehicle, 0.01 mg/L CBN, 0.1 mg/L CBN, 0.5 mg/L CBN, 1 mg/L CBN, 2 mg/L CBN, 3 mg/L CBN and 4 mg/L CBN during gastrulation (N = 4 experiments and n = 20 embryos for each treatment). Groups which share the same letters are not significantly different from each other.

**Figure 2: Dose response effects of Cb1r and Cb2r antagonists (AM251 and CP945598) during gastrulation.** (**A**) Images of embryos exposed to DMSO (vehicle control), 5 nM AM251, 10 nM AM251, 20 nM AM251, 50 nM AM251, 100 nM AM251, and 5 nM CP945598, 10 nM CP945598, 20 nM CP945598, 50 nM CP945598, 100 nM CP945598 (from 5.25 hpf to 10.75 hpf) and then allowed to develop in normal embryo media. Images were taken at 48–52 hpf. (**B**) Images of embryos exposed to DMSO (vehicle control), 5 µM AM630, 10 µM AM630, 20 µM AM630, 50 µM AM630, 100 µM AM630, and 5 µM JTE907, 10 µM JTE907, 20 µM JTE907, 50 µM JTE907, 100 µM JTE907 (from 5.25 hpf to 10.75 hpf) and then allowed to develop in normal embryo media. Images were taken at 48–52 hpf. (C-E) Bar graph showing the body lengths (in mm) of embryos (C), % embryos with curved tails (D) and % embryos with edema (E) exposed to different media: DMSO (vehicle control) (n = 60), 5 nM AM251 (n = 60), 10 nM AM251 (n = 60), 20 nM AM251 (n = 55), 50 nM AM251 (n = 40), 100 nM AM251 (n = 30) and 5 nM CP945598 (n = 60), 10 nM CP945598 (n = 68), 20 nM CP945598 (n = 66), 50 nM CP945598 (n = 42), 100 nM CP945598 (n = 40), respectively at 2 dpf. (F-H) Bar graph showing the body lengths (in mm) of embryos (F), % embryos with curved tails (G) and % embryos with edema (H) exposed to different media: DMSO (vehicle control) (n = 50), 5 µM AM630 (n = 50), 10 µM AM630 (n = 40), 20 µM AM630 (n = 50), 50 µM AM630 (n = 40), 100 µM AM630 (n = 40) and 5 µM JTE907 (n = 70), 10 µM JTE907 (n = 68), 20 µM JTE907 (n = 60), 50 µM JTE907 (n = 52), 100 µM JTE907 (n = 30), respectively at 2 dpf. Groups which share the same letters are not significantly different from each other.

**Figure 3: Effect of Cb1r antagonists on survival and hatching.** (A) Line graphs showing the percentage of embryos that survived within the first 5 days of development following exposure to DMSO (vehicle control) or AM251 (5-100 nM). (B) Line graphs showing the percentage of embryos that survived within the first 5 days of development following exposure to DMSO (vehicle control) or CP945598 (5-100 nM). (C) Line graphs showing the percentage of embryos that survived within the first 5 days of development following exposure to DMSO (vehicle control) or AM630 (5-100 µM). (D) Line graphs showing the percentage of embryos that survived within the first 5 days of development following exposure to DMSO (vehicle control) or JTE907 (5-100 µM). (E) Line graphs showing the percentage of embryos that hatched within the first 5 days of development following exposure to DMSO (vehicle control) or AM251 (5-100 nM). (F) Line graphs showing the percentage of embryos that hatched within the first 5 days of development following exposure to DMSO (vehicle control) or CP945598 (5-100 nM). (G) Line graphs showing the percentage of embryos that hatched within the first 5 days of development following exposure to DMSO (vehicle control) or AM630 (5-100 µM). (H) Line graphs showing the percentage of embryos that hatched within the first 5 days of development following exposure to DMSO (vehicle control) or JTE907 (5-100 µM). Groups which share the same letters are not significantly different from each other.

**Figure 4: Effect of Cb1r antagonist on locomotion.** The free swimming movements of 5 dpf larval zebrafish was recorded for 60 min and the recorded video was later analyzed. Recordings were made for the following treatments: DMSO (vehicle control), AM251 (5-100 nM or CP945598 (5-100 nM) (A, B). (A) Bar graph shows the total distance swam (mm) for 1 h, (B) Bar graph shows the mean activity of swimming as a percentage of time. Groups which share the same letters are not significantly different from each other. (C, D) Recordings were made for the following treatments: DMSO (vehicle control), AM630 (5-10 µM) or JTE907 (5-100 µM). (C) Bar graph shows the total distance swam (mm) for 1 h, (D) Bar graph shows the mean activity of swimming as a percentage of time. Groups which share the same letters are not significantly different from each other.

**Figure 5: Raw tracing of primary MN.** Neurite image tracing (Image J) was used to trace the MN branching of confocal images. (A) Tracing showed for vehicle control, (B) 3 mg/L CBN, (C) 3 mg/L CBN + 10 nM AM251, (D) 3 mg/L CBN + 10 µM AM630 and (E) 3 mg/L CBN + 10 nM AM251 + 10 µM AM630. Otolith images were taken from 52 hpf old embryos, positioning anterior (head) on right and posterior (tail) on left. (F) 3 mg/L CBN, (G) 3 mg/L CBN + 10nM AM251, (H) 3 mg/L CBN + 10 µM AM630 and (I) 3 mg/L CBN + 10 nM AM251 + 10 µM AM630. Scale bar represents 50 µm for F-I.

**Supplement Video 1:** Cardiac activity in vehicle control embryo. Video was recorded at 2dpf at 10x magnification.

**Supplement Video 2:** Cardiac activity in embryo treated with 3 mg L^-1^ CBN. Video was recorded at 2dpf at 10x magnification.

**Supplement Video 3:** Cardiac activity in embryo treated with 3 mg L^-1^ CBN + 10 µM AM630. Video was recorded at 2dpf at 10x magnification.

**Supplement Video 4:** 3D reconstruction of motor neuron branching in vehicle control embryos. Videos were prepared using Imaris software.

**Supplement Video 5:** 3D reconstruction of motor neuron branching in embryos treated with 3 mg L^-1^ CBN. Videos were prepared using Imaris software.

**Supplement Video 6:** 3D reconstruction of motor neuron branching in embryos treated with 3 mg L^-1^ CBN + 10 µM AM630. Videos were prepared using Imaris software.
